# Supplementary material for: Do Single-Nucleotide Polymorphisms Affect Pain Intensity and Sufentanil Analgesia After Pediatric Scoliosis Correction Surgery?
Source: Int J Mol Sci. 2025 Apr 9;26(8):3504. doi: 10.3390/ijms26083504 (PMC12026534; doi:10.3390/ijms26083504)
Supplement: Supplementary file 1 [file ijms-26-03504-s001.zip › ijms-3554113-supplementary.pdf]

Table S1. Outcomes of regression models created to indicate the influence of polymorphism on clinical variables

Polymorphism		CRP 72h post-surgery			Mean 24h NRS Pain Rating			Sufentanil mean infusion flow rate-weight (adjusted)			PACU length-of-stay			Coanalgesics (yes/no)		
		**β (95% CI)**	**p**	**p adj**	**β (95% CI)**	**p**	**p adj**	**β (95% CI)**	**p**	**p adj**	**β (95% CI)**	**p**	**p adj**	**OR (95% CI)**	**p**	**p adj**
rs1799971 OPRM1	AA	ref			ref			ref			ref			ref		
	AG	-32.12 (-84.85;20.61)	0.225	0.390	-0.26 (-1.28;0.76)	0.612	0.663	-0.01 (-0.03;0.01)	0.410	0.662	-0.28 (-0.95;0.39)	0.405	0.911	6.35 (0.95;126.67)	0.102	0.544
rs1205 CRP	CC	ref			ref			ref			ref			ref		
	CT	-9.76 (-53.00;33.49)	0.650	0.761	0.25 (-0.57;1.08)	0.542	0.613	0.00 (-0.02;0.02)	0.837	0.907	-0.62 (-1.10;-0.14)	**0.012**	0.068	0.70 (0.18;2.57)	0.592	0.861
	TT	0.67 (-69.15;70.49)	0.985	0.985	0.06 (-1.22;1.34)	0.929	0.929	-0.01 (-0.04;0.03)	0.660	0.817	0.03 (-0.71;0.77)	0.927	0.999	1.05 (0.14;9.61)	0.962	0.999
rs1045642 ABCB1	AA	ref			ref			ref			ref			ref		
	AG	-12.66 (-62.23;36.91)	0.608	0.753	-0.37 (-1.33;0.58)	0.433	0.613	-0.01 (-0.03;0.01)	0.433	0.662	0.51 (-0.08;1.09)	0.087	0.241	0.51 (0.11;2.29)	0.386	0.861
	GG	-17.05 (-78.41;44.31)	0.577	0.750	-0.44 (-1.60;0.72)	0.451	0.613	-0.02 (-0.05;0.01)	0.117	0.234	0.72 (0.01;1.43)	**0.046**	0.138	2.33 (0.33;21.56)	0.410	
rs6269	AA	ref			ref			ref			ref			ref		
	AG	9.41 (-35.39;54.20)	0.673	0.761	-0.27 (-1.12;0.58)	0.529	0.613	-0.01 (-0.03;0.01)	0.527	0.721	0.05 (-0.50;0.61)	0.843	0.999	0.67 (0.17;2.50)	0.556	0.861
rs4633	CC	ref			ref			ref			ref			ref		
	CT	48.04 (-9.93;106.01)	0.102	0.189	0.68 (-0.46;1.81)	0.234	0.435	0.01 (-0.02;0.03)	0.638	0.817	0.02 (-0.72;0.76)	0.963	0.999	0.72 (0.09;4.58)	0.737	0.917

	TT	20.56 (-41.83;82.95)	0.509	0.697	0.45 (-0.77;1.68)	0.461	0.613	0.01 (-0.02;0.04)	0.372	0.645	-0.21 (-0.99;0.57)	0.590	0.999	0.38 (0.04;2.61)	0.337	0.861
rs4818	CC	ref			ref			ref			ref			ref		
	CG	18.59 (-21.05;58.22)	0.349	0.534	-0.64 (-1.40;0.11)	0.091	0.350	0.00 (-0.02;0.02)	0.941	0.941	0.21 (-0.28;0.70)	0.395	0.911	0.30 (0.08;1.03)	0.063	0.544
rs4680	AA	ref			ref			ref			ref			ref		
	AG	46.24 (4.03;88.45)	**0.033**	0.078	0.34 (-0.55;1.22)	0.447	0.613	0.00 (-0.02;0.02)	0.830	0.907	-0.03 (-0.59;0.53)	0.914	0.999	1.22 (0.33;4.65)	0.765	0.917
	GG	-21.04 (-78.38;36.31)	0.462	0.667	-0.42 (-1.59;0.76)	0.479	0.613	0.00 (-0.03;0.03)	0.905	0.941	0.00 (-0.78;0.79)	0.991	0.999	2.00 (0.30;17.59)	0.488	0.861
COMT haplotype	ACCA/GTGG	ref			ref			ref			ref			ref		
	ATCA/ATCA	-48.74 (-101.52;4.03)	0.069	0.150	-0.10 (-1.18;0.99)	0.857	0.891	0.00 (-0.03;0.02)	0.746	0.882	0.06 (-0.62;0.75)	0.856	0.999	1.60 (0.30;8.90)	0.581	0.861
	Other	-38.30 (-83.97;7.37)	0.098	0.189	-0.29 (-1.20;0.62)	0.523	0.613	-0.02 (-0.04;0.00)	0.115	0.234	0.02 (-0.56;0.61)	0.932	0.999	3.47 (0.82;16.32)	0.099	0.544
rs7832704 ENPP2	AA	ref			ref			ref			ref			ref		
	AG	-1.93 (-65.13;61.28)	0.951	0.985	-0.56 (-1.74;0.62)	0.342	0.593	0.02 (-0.01;0.05)	0.234	0.435	-0.26 (-1.01;0.49)	0.488	0.999	0.78 (0.09;5.27)	0.802	0.917
rs1801253 ADRB1	CC	ref			ref			ref			ref			ref		
	CG	-20.26 (-63.27;22.75)	0.346	0.534	-0.53 (-1.34;0.29)	0.199	0.398	-0.01 (-0.03;0.01)	0.521	0.721	-0.15 (-0.69;0.39)	0.581	0.999	1.80 (0.48;7.26)	0.389	0.861

GG	12.78 (-82.64;108.20)	0.788	0.854	-1.92 (-3.66;-0.17)	**0.032**	0.350	-0.04 (-0.08;0.01)	0.093	0.234	0.86 (-0.30;2.02)	0.140	0.360	1.00 (0.04;27.14)	>0.999	0.999

β – model estimate from linear regression (all clinical parameters except from Coanalgesics) expressing the relationship between polymorphism and clinical parameter, OR – odds ratio based on logistic regression (Coanalgesics) expressing the relationship between Polymorphism and usage of Coanalgesics, CI – confidence interval, p adj – p values after applying Benjamini & Hochberg correction. All models assumed clinical parameter as dependent variable and polymorphism as independent variable with additional confounder treated as independent variable. Age was confounder for CRP 72h post-surgery and Sufentanil mean infusion flow rate-weight (adjusted), sex was confounder for Mean 24h NRS Pain Rating and type of procedure was confounder for PACU length-of-stay. Models for Coanalgesics do not include any confounder due to all potential confounders with p>0.250 in univariate analysis (table 2).
